# 

**Published:** 1850-10

**Authors:** 


### CONTENTS

### OF NO. IV.

---

### ORIGINAL COMMUNICATIONS.

### ESSAYS AND CASES.

Art. I.—A Report on Typhoid Fever made to the Scott County Medical Society. By W. L. Sutton, M. D., of Georgetown, Ky. - - - - - - - 277
Art. II.—On Watering Streets. By Theodore S. Bell, M. D., of Louisville, Ky. - - - - - 327
Art. III.—Jarvis's Adjuster for the Reduction of Dislocations, and Treatment of Fractures. By Theodore S. Bell, M. D., of Louisville, Ky. - - - - 331

### REVIEWS.

Art. IV.—A Systematic Treatise, Historical, Etiological, and Practical, on the Principal Diseases of the Interior Valley of North America, as they appear in the Caucasian, African, Indian, and Esquimaux varieties of its Population. By Daniel Drake, M. D. - - 350

### ORIGINAL INTELLIGENCE.

University of Louisville, - - - - - - 361
Prolonged Lecture Terms, - - - - - - 362

The New York Register of Medicine and Pharmacy. Edited by C. D. Griswold, M. D. - - - - - - 363
The Western Medico-Chirurgical Journal. Edited by J. F. Sanford, M. D., and Samuel G. Armor, M. D., Professors in the Iowa State University, - - - 363
Medical Teaching in New York, - - - - - 364
Practical Medicine, - - - - - - - 365
Anatomical Drawings, - - - - - - 365
The Western Lancet, and the Memphis School Advertisement, 366
The Memphis School, - - - - - - 366
Chloroform, - - - - - - - 366
The Largest Liberty, - - - - - - 367

